# Fourier and microlocal methods for quantum Rabi systems

**DOI:** 10.1007/s11565-026-00749-7

**Published:** 2026-09-21

**Authors:** Marcello Malagutti, Alberto Parmeggiani

**Affiliations:** 1 Mathematics Institute, University of Warwick, Coventry, CV4 7AL UK; 2 Dipartimento di Matematica, Università di Bologna, Piazza di Porta San Donato 5, 40126 Bologna, Italy

**Keywords:** Quantum Rabi model, Fourier analysis, Global pseudodifferential calculus, Analytic perturbation theory, Braak conjecture, Weyl law, Primary 35P20, Secondary 35S05, 81Q10

## Abstract

We give a concise presentation two perturbative approaches to the spectral analysis of Quantum Rabi systems. For the one-mode two-level model, a metaplectic translation reduces the Hamiltonian to a bounded analytic perturbation of a doubled harmonic oscillator. The first-order effective matrix is computed by Fourier-Hermite methods and its entries are expressed through Laguerre polynomials. This gives analytic spectral branches, parity information, and the validity of Braak’s conjecture on arbitrary fixed finite spectral windows for a sufficiently small atomic gap. We also discuss multi-level, multi-mode Rabi systems, for n=2 and N=3. Although their semiprincipal symbols need not be smoothly diagonalizable, an order-one regularizing perturbation places them in a globally diagonalizable class; comparison by the minimax principle then yields a two-term Weyl asymptotic formula. Complete proofs and additional computations appear in [12]; here the emphasis is on the Fourier and microlocal techniques behind the results.

## Introduction

The Quantum Rabi model is the basic non-rotating-wave Hamiltonian describing the interaction of a two-level quantum system with one quantized bosonic mode. In suitable units it is written

1 $$\begin{aligned} Q_{\textrm{Rabi}} =\boldsymbol{a}^\dagger \boldsymbol{a}\,I_2 +g(\boldsymbol{a}^\dagger +\boldsymbol{a})\sigma _x +\Delta \sigma _z, \end{aligned}$$

where $\boldsymbol{a}$ and $\boldsymbol{a}^\dagger$ are the annihilation and creation operators, 2Δ is the atomic level splitting, and g>0 is the coupling strength. In contrast with the Jaynes–Cummings model, the counter-rotating terms are retained. The Hamiltonian preserves a $\mathbb {Z}_2$ parity, but it does not preserve the excitation number which makes its spectral geometry both rigid and nontrivial [1, 2, 6].

The purpose of this paper is to isolate two analytic approaches that are especially natural from the point of view of Fourier and microlocal analysis.

The first approach is based on a perturbative study of the Quantum Rabi model. The perturbative parameter in this case is the atomic energy gap. After a fixed change of basis and a phase-space translation, the one-dimensional Rabi Hamiltonian becomes a bounded analytic perturbation of the scalar harmonic oscillator acting on $L^2(\mathbb {R};\mathbb {C}^2)$. The two-dimensional eigenspaces of the unperturbed operator are treated by Rellich theory. The corresponding effective matrix has off-diagonal entries

$$ (T_+\varphi _N,T_-\varphi _N)_{L^2}, $$

where $T_\pm$ are translations and $\varphi _N$ is a Hermite eigenfunction. A Fourier-Hermite computation identifies this overlap with a Laguerre polynomial. Thus the geometry of the first-order spectral splitting is determined by the zeros of a classical family of orthogonal polynomials. Second, the high-energy analysis of multi-level and multi-mode Rabi systems can be carried out by using the global Weyl calculus. The principal symbol is scalar, but the order-one part fails to admit a smooth global diagonalization. We add an order-one pseudodifferential perturbation which restores global diagonalizability, apply the Weyl law for semiregular metric globally elliptic systems (a class of principally scalar pseudodifferential systems) developed in [10], and then remove the auxiliary perturbation by a quadratic-form comparison and the minimax principle. We then show how the result can be applied to the generalizations of the Rabi system in dimension n=2 and size N=3, which represent interactions of two photons with a three-level atom.

Related spectral results for semiregular non-commutative harmonic oscillators include the analysis of the spectral zeta function in [9] and quasi-clustering estimates in [11].

The results in this paper are a concise presentation of results proved in full detail in [12], focusing on the method developed and used there. In particular, whenever a proof is reduced to its main steps, the precise estimates, convergence statements, and supplementary calculations can be found there. Namely, here we focus on the role of metaplectic covariance, Hermite expansions, analytic spectral projections, and global pseudodifferential methods.

The principal perturbative conclusion for the one-mode Rabi model can be summarized as follows (see Conjecture 3.5 for the statement of the Braak conjecture).

### Theorem 1.1

(Finite-window form of Braak’s conjecture) For every $k_0\in \mathbb {N}$ there exists $\varepsilon _0>0$ such that, for every $N\le k_0$ and $0<|\varepsilon |\le \varepsilon _0$, Braak’s conjecture holds for the shifted perturbed Rabi Hamiltonian $\widehat{Q}_{R,\varepsilon }$ (see (11) below) in each interval [N+1/2,N+3/2). Moreover,

$$ \#\bigl (\operatorname {Spec}\widehat{Q}_{R,\varepsilon } \cap [N+1/2,N+3/2)\bigr )\in \{1,2,3\}. $$

The statement includes both generic couplings, where the double harmonic-oscillator eigenvalues split at first order, and exceptional couplings, where the first-order effective matrix is scalar. In the latter case the eigenvalues may split only at higher order, but parity and analytic spectral projections still give enough information for Braak’s counting statement.

For generalized *N*-level systems, with n=N-1 bosonic modes, the main asymptotic conclusion is the following. Here $p_2(X)=|X|^2/2$ on $\mathbb {R}^{2n}$ (where X=(x,ξ)).

### Theorem 1.2

(Weyl asymptotics) Let $A=a^{\mathrm w}(x,D)$ be a positive self-adjoint globally elliptic N×N system with semiregular symbol

$$ a\sim a_2+a_1+a_0+\cdots ,\qquad a_2=p_2I_N. $$

Assume that there exists a self-adjoint, positively homogeneous (in *X*) symbol $b_1$ of degree one such that $a+\varepsilon b_1$ is a semiregular metric globally elliptic system (SMGES) for every $\varepsilon \in (0,1)$. (See [10] for the definition of SMGES.) Then

2 $$\begin{aligned} \mathsf N_A(\lambda ) =(2\pi )^{-n}\bigg (&N\lambda ^n\int _{p_2\le 1}dX\nonumber \\&-\lambda ^{n-1/2}\int _{p_2=1}\operatorname {Tr}(a_1) \frac{ds}{|\nabla p_2|}\bigg )+o(\lambda ^{n-1/2}). \end{aligned}$$

The paper is arranged as follows. Section 2 introduces the global calculus and the unitary normal form. Section 3 treats the non-degenerate first-order splitting, parity, and Braak’s conjecture. Section 4 discusses exceptional couplings and higher-order splitting. Section 5 gives the microlocal regularization and the Weyl comparison argument.

## Global framework and a unitary normal form

### A minimal global pseudodifferential setup

We write $X=(x,\xi )\in T^*\mathbb {R}^n\simeq \mathbb {R}^{2n}$ and $\left\langle X\right\rangle =(1+|X|^2)^{1/2}$. A smooth function *a* belongs to the Shubin class $S^m_{\textrm{iso}}(\mathbb {R}^n)$ if

3 $$\begin{aligned} |\partial _X^\gamma a(X)|\le C_\gamma \left\langle X\right\rangle ^{m-|\gamma |} \quad \text {for every multi-index }\gamma . \end{aligned}$$

Its Weyl quantization is

4 $$\begin{aligned} a^{\mathrm w}(x,D)u(x) =(2\pi )^{-n}\iint _{\mathbb {R}^{2n}}e^{i\langle x-y,\xi \rangle } a\left( \frac{x+y}{2},\xi \right) u(y)\,dy\,d\xi . \end{aligned}$$

We use the same notation for matrix-valued symbols. A semiregular symbol has an asymptotic expansion in positively homogeneous terms,

$$ a\sim a_m+a_{m-1}+a_{m-2}+\cdots . $$

A self-adjoint semiregular symbol is called a semiregular metric globally elliptic system (SMGES) when its principal symbol is scalar and globally elliptic and its semiprincipal symbol is smoothly unitarily diagonalizable, away from the origin, into spectrally ordered blocks. We refer to [4, 5, 10, 13, 16] for the calculus and the spectral consequences of this condition.

The Weyl calculus is covariant under affine symplectic transformations. In fact, if χ is affine symplectic, there is a metaplectic operator $U_\chi$ such that

5 $$\begin{aligned} U_\chi ^*a^{\mathrm w}(x,D)U_\chi =(a\circ \chi )^{\mathrm w}(x,D). \end{aligned}$$

For the Rabi model, this elementary form of Egorov covariance is the key result: the linear interaction can be absorbed by translating the harmonic oscillator wells.

### The one-dimensional Rabi system

Let

$$ p_2(x,\xi )=\frac{x^2+\xi ^2}{2},\qquad P_0=p_2^{\mathrm w}(x,D)=\frac{D_x^2+x^2}{2}. $$

The spectrum of $P_0$ is $\{N+1/2:N\in \mathbb {Z}_+\}$, with Hermite eigenfunctions

6 $$\begin{aligned} \varphi _N(x)=\left( \frac{x-\partial _x}{\sqrt{2}}\right) ^Ne^{-x^2/2} =H_N(x)e^{-x^2/2}, \qquad \Vert \varphi _N\Vert _0^2=\sqrt{\pi }\,N!. \end{aligned}$$

We consider

7 $$\begin{aligned} Q_{R,\varepsilon } =P_0I_2+\alpha x\sigma _x +\varepsilon \begin{bmatrix}\gamma _1& 0\\ 0& \gamma _2\end{bmatrix}, \qquad \alpha \ne 0,\quad \gamma _1>\gamma _2, \end{aligned}$$

with maximal domain $B^2(\mathbb {R};\mathbb {C}^2)$. It is self-adjoint and has compact resolvent. The genuine Rabi Hamiltonian is obtained by taking $\gamma _1=-\gamma _2=\Delta$ and subtracting $I_2/2$.

Let

$$ T_\pm u(x)=u(x\mp \alpha ), \qquad U_0=\frac{1}{\sqrt{2}}\begin{bmatrix}1& 1\\ 1& -1\end{bmatrix}, \qquad T=\begin{bmatrix}T_+& 0\\ 0& T_-\end{bmatrix}. $$

#### Lemma 2.1

(Unitary normal form) There is a unitary metaplectic operator $U=U_0T^*$ such that

8 $$\begin{aligned} U^*\left( Q_{R,\varepsilon }+\frac{\alpha ^2}{2}I_2\right) U =A+\varepsilon B, \end{aligned}$$

where

$$ A=P_0I_2, \qquad B=\begin{bmatrix} \beta _1& \beta _2T_+^2\\ \beta _2T_-^2& \beta _1 \end{bmatrix}, \qquad \beta _1=\frac{\gamma _1+\gamma _2}{2},\quad \beta _2=\frac{\gamma _1-\gamma _2}{2}. $$

#### Proof

Conjugation by $U_0$ diagonalizes $\sigma _x$ and transforms the last matrix in (7) into $$\bigl [{\begin{smallmatrix}\beta _1& \beta _2\\ \beta _2& \beta _1\end{smallmatrix}}\bigr ]$$. Completing the square gives

$$\begin{aligned} U_0^*\left( Q_{R,\varepsilon }+\frac{\alpha ^2}{2}I_2\right) U_0 ={}&\begin{bmatrix} \frac{1}{2}(D^2+(x+\alpha )^2)& 0\\ 0& \frac{1}{2}(D^2+(x-\alpha )^2) \end{bmatrix}\\&+\varepsilon \begin{bmatrix}\beta _1& \beta _2\\ \beta _2& \beta _1\end{bmatrix}. \end{aligned}$$

The two diagonal oscillators are translated back to $P_0$ by $T_+$ and $T_-$, respectively. This yields (8). □

Since the translations are unitary, *B* is bounded and self-adjoint on $L^2(\mathbb {R};\mathbb {C}^2)$. Hence $\varepsilon \mapsto A+\varepsilon B$ is an analytic family of type (A), with constant domain $B^2(\mathbb {R};\mathbb {C}^2)$, and every isolated finite cluster of eigenvalues and its Riesz projection depend analytically on ε [7, 14].

### Relation with the usual Rabi Hamiltonian

With the convention

$$ \boldsymbol{a}=\frac{x+\partial _x}{\sqrt{2}},\qquad \boldsymbol{a}^\dagger =\frac{x-\partial _x}{\sqrt{2}}, $$

one has $\boldsymbol{a}^\dagger \boldsymbol{a}=P_0-1/2$ and $\boldsymbol{a}+\boldsymbol{a}^\dagger =\sqrt{2}x$. Thus, after putting $g=\alpha /\sqrt{2}$ and replacing the atomic splitting by $\varepsilon \Delta$, the usual Rabi Hamiltonian is

9 $$\begin{aligned} Q_{\textrm{Rabi},\varepsilon } =P_0I_2+\alpha x\sigma _x+\varepsilon \Delta \sigma _z-\frac{1}{2}I_2. \end{aligned}$$

It follows that

$$ Q_{R,\varepsilon }=Q_{\textrm{Rabi},\varepsilon }+\frac{1}{2}I_2 $$

when $\gamma _1=-\gamma _2=\Delta$. Under the unitary transformation of Lemma 2.1,

10 $$\begin{aligned} U^*Q_{\textrm{Rabi},\varepsilon }U =P_0I_2+\varepsilon \Delta \begin{bmatrix}0& T_+^2\\ T_-^2& 0\end{bmatrix} -\frac{1+\alpha ^2}{2}I_2. \end{aligned}$$

The additive scalar shifts do not affect multiplicity or parity since they only determine which unit intervals are used in the formulation of Braak’s conjecture.

Since Braak’s conjecture deals with the shifted eigenvalues of the Quantum Rabi model, we introduce for convenience the **shifted perturbed Quantum Rabi Hamiltonian operator**

11 $$\begin{aligned} \widehat{Q}_{R,\varepsilon }=Q_{R,\varepsilon }+ \frac{\alpha ^2}{2}I_2=Q_{\textrm{Rabi},\varepsilon }+\frac{1+\alpha ^2}{2}\,I_2. \end{aligned}$$

The normal form also clarifies the Fourier content of the perturbation. If $\mathcal {F}$ is the unitary Fourier transform, then

$$ \mathcal {F} T_\pm ^2\mathcal {F}^{-1} =e^{\mp 2i\alpha \xi }. $$

Hence the transformed interaction is a bounded matrix Fourier multiplier. The perturbation is nonlocal in the position variable, but its action on oscillator eigenspaces is explicitly computable because Hermite functions are Fourier eigenfunctions.

For the genuine Rabi model, the parity operator is

12 $$\begin{aligned} \Pi =(-1)^{\boldsymbol{a}^\dagger \boldsymbol{a}}\sigma _z. \end{aligned}$$

It commutes with $Q_{R,\varepsilon }$ and gives the orthogonal decomposition

$$ L^2(\mathbb {R};\mathbb {C}^2)=\boldsymbol{H}_+\oplus \boldsymbol{H}_-, $$

where

$$\begin{aligned} \boldsymbol{H}_+&=\{(f_1,f_2):f_1\text { even},\ f_2\text { odd}\},\\ \boldsymbol{H}_-&=\{(f_1,f_2):f_1\text { odd},\ f_2\text { even}\}. \end{aligned}$$

## First-order splitting and Braak’s conjecture

### The effective matrix

Fix $N\in \mathbb {Z}_+$ and put $\lambda _N=N+1/2$. The eigenspace

$$ E_N=\operatorname {Ker}(A-\lambda _N) =\operatorname {span}\{\phi _N\otimes e_1,\phi _N\otimes e_2\}, \qquad \phi _N=\varphi _N/\Vert \varphi _N\Vert _0, $$

has dimension two. The first Rellich matrix is the compression of *B* to $E_N$:

13 $$\begin{aligned} M_N= \begin{bmatrix} \beta _1& \beta _2c_N\\ \beta _2c_N& \beta _1 \end{bmatrix}, \qquad c_N=\frac{(T_+\varphi _N,T_-\varphi _N)_0}{\Vert \varphi _N\Vert _0^2}. \end{aligned}$$

Its eigenvalues are

14 $$\begin{aligned} \mu _{N,\pm }=\beta _1\pm \beta _2|c_N|. \end{aligned}$$

Thus the double eigenvalue $\lambda _N$ splits at first order precisely when $c_N\ne 0$.

The coefficient $c_N$ has a simple Fourier-Hermite description. The translation $T_\pm$ becomes multiplication by $e^{\mp i\alpha \xi }$ under the Fourier transform, while the Hermite functions diagonalize the Fourier transform. Equivalently, one can use the translation identity

$$ H_N(x+y)=\sum _{j=0}^N\left( {\begin{array}{c}N\\ j\end{array}}\right) H_j(x)(\sqrt{2}y)^{N-j} $$

and Hermite orthogonality. Both routes lead to the following formula.

#### Lemma 3.1

(Hermite–Laguerre overlap) For $N,k\in \mathbb {Z}_+$,

15 $$\begin{aligned} (T_+\varphi _N,T_-\varphi _k)_0 =e^{-\alpha ^2} \sum _{j=0}^{\min \{N,k\}} (-1)^{N-j}\left( {\begin{array}{c}N\\ j\end{array}}\right) \left( {\begin{array}{c}k\\ j\end{array}}\right) (\sqrt{2}\alpha )^{N+k-2j}\Vert \varphi _j\Vert _0^2. \end{aligned}$$

In particular,

16 $$\begin{aligned} (T_+\varphi _N,T_-\varphi _N)_0 =\sqrt{\pi }\,e^{-\alpha ^2}N!L_N(2\alpha ^2), \end{aligned}$$

up to the harmless sign convention determined by the chosen definition of $L_N$.

#### Idea of the proof

Insert the translation formula for both Hermite polynomials in

$$ e^{-\alpha ^2}\int _\mathbb {R}e^{-x^2}H_N(x-\alpha )H_k(x+\alpha )\,dx. $$

Orthogonality eliminates all terms with different Hermite indices. For k=N, the remaining binomial sum is precisely the Laguerre expansion. See [12, Section 4] for the detailed normalization and sign bookkeeping. □

Let

17 $$\begin{aligned} \mathsf Z=\{x\ge 0:L_k(x)=0\text { for some }k\ge 0\}. \end{aligned}$$

The zeros of the Laguerre polynomials are positive, simple, and interlace; the union Z is countable and dense in [0,∞) (see [3]). For $2\alpha ^2\notin \mathsf Z$, every fixed finite collection of overlaps is uniformly separated from zero.

The following elementary compactness statement is the precise form needed below.

#### Lemma 3.2

(Separation on finite spectral windows) Let $x_0\notin \mathsf Z$. For every $K\in \mathbb {N}$ there exists $\delta _K>0$ such that

$$ \operatorname {dist}\bigl (x_0,L_N^{-1}(0)\bigr )\ge \delta _K, \qquad 0\le N\le K. $$

Equivalently, there exists $m_K>0$ such that

$$ |L_N(x_0)|\ge m_K, \qquad 0\le N\le K. $$

Moreover, one may choose a sequence $K_j\rightarrow \infty$ and corresponding $\delta _j\downarrow 0$ with the same property for all $N\le K_j$.

#### Proof

For fixed *K*, the union of the zero sets of $L_0,\ldots ,L_K$ is finite and does not contain $x_0$; its distance from $x_0$ is therefore positive. The second assertion follows by continuity and finiteness. To construct increasing windows, take a decreasing sequence of neighborhoods of $x_0$ and let $K_j$ be the first polynomial index having a zero in the *j*-th neighborhood. Passing to a subsequence gives $K_j\rightarrow \infty$ and $\delta _j\downarrow 0$. This is the form used in the full Rellich construction; see [12, Lemma 4.2]. □

### Analytic branches

#### Theorem 3.3

(Non-degenerate finite clusters) Assume $2\alpha ^2\notin \mathsf Z$. For every $K\in \mathbb {N}$ there exists $c_K>0$ such that, for $0\le N\le K$, there are real-analytic eigenvalue and eigenvector branches

$$ \varepsilon \longmapsto \lambda _{N,\pm }(\varepsilon ), \qquad \varepsilon \longmapsto u_{N,\pm }(\varepsilon ), \qquad |\varepsilon |<c_K, $$

satisfying

18 $$\begin{aligned} (A+\varepsilon B)u_{N,\pm }(\varepsilon )&=\lambda _{N,\pm }(\varepsilon )u_{N,\pm }(\varepsilon ),\end{aligned}$$

19 $$\begin{aligned} \lambda _{N,\pm }(\varepsilon )&=N+\frac{1}{2}+\mu _{N,\pm }\varepsilon +O(\varepsilon ^2), \end{aligned}$$

with $\Vert u_{N,\pm }(\varepsilon )\Vert _0=1$. For $\varepsilon \ne 0$ sufficiently small, all these eigenvalues are simple.

#### Proof sketch

The family A+εB is analytic of type (A). The restriction of the first derivative to $E_N$ is $M_N$. By (16) and the assumption on α, the two eigenvalues of $M_N$ are distinct. Rellich’s construction therefore produces two analytic eigenvalue branches and corresponding analytic normalized eigenvectors. The minimum first-order separation over $0\le N\le K$ is positive, so a single $c_K$ works for the whole finite cluster. Elliptic regularity shows that every coefficient in the eigenvector expansion is a Schwartz function. Full details are given in [12, Section 4]. □

The parity of the branches is also explicit. Put

$$ s_N=\operatorname {sgn}(T_+\varphi _N,T_-\varphi _N)_0, \qquad \sigma _N=(-1)^Ns_N. $$

#### Proposition 3.4

(Parity of the non-degenerate branches) The eigenvalue $\lambda _{N,\pm }(\varepsilon )$ of $\widehat{Q}_{R,\varepsilon }$ has parity $\pm \sigma _N$ for every sufficiently small ε.

#### Idea of the proof

At ε=0, eigenvectors of $M_N$ are proportional to $(s_N,\pm 1)$. Conjugating them back through $U=U_0T^*$ gives combinations of $T_-\varphi _N$ and $T_+\varphi _N$. Since $\varphi _N(-x)=(-1)^N\varphi _N(x)$, these combinations belong to $\boldsymbol{H}_{\pm \sigma _N}$. For $\varepsilon \ne 0$, simplicity forces an eigenvector to lie in one parity subspace; continuity prevents the parity from changing along the analytic branch. □

### Braak’s counting statement

We use the formulation in terms of parity-resolved counts. Set

$$ B_N^\pm =\#\left( \operatorname {Spec}(\widehat{Q}_{R,\varepsilon } |_{\boldsymbol{H}_\pm }) \cap [N+1/2,N+3/2)\right) . $$

#### Conjecture 3.5

(Braak) For every N≥0 and each parity,

$$ B_N^\pm \in \{0,1,2\}, \qquad B_N^\pm +B_{N+1}^\pm \in \{1,2,3\}. $$

**Fig. 1:**
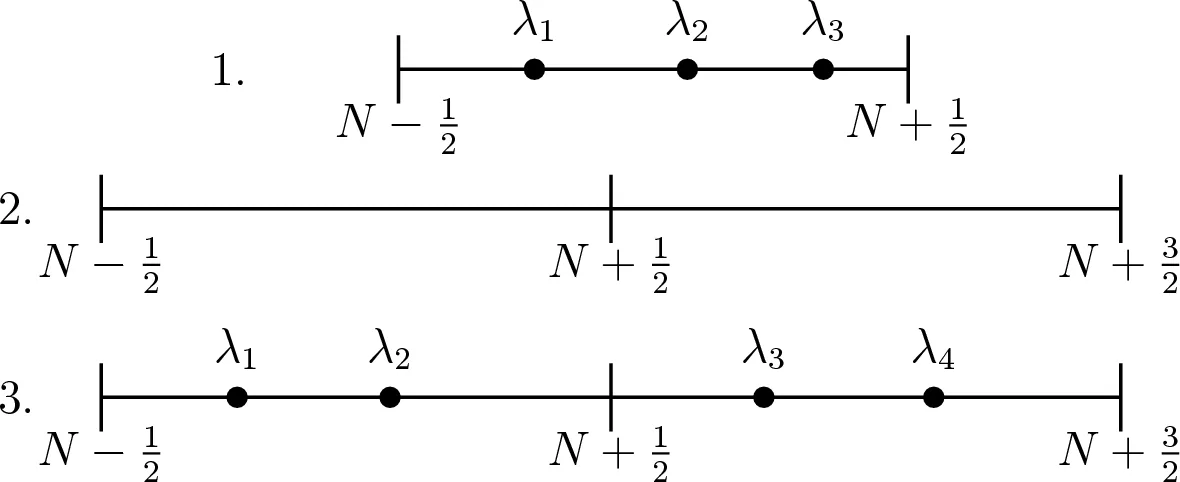
Braak’s conjecture is false if for a given parity: 1. There are more than two eigenvalues in an interval; 2. There are two adjacent intervals without eigenvalues; 3. There are two adjacent intervals with two eigenvalues

**Fig. 2:**
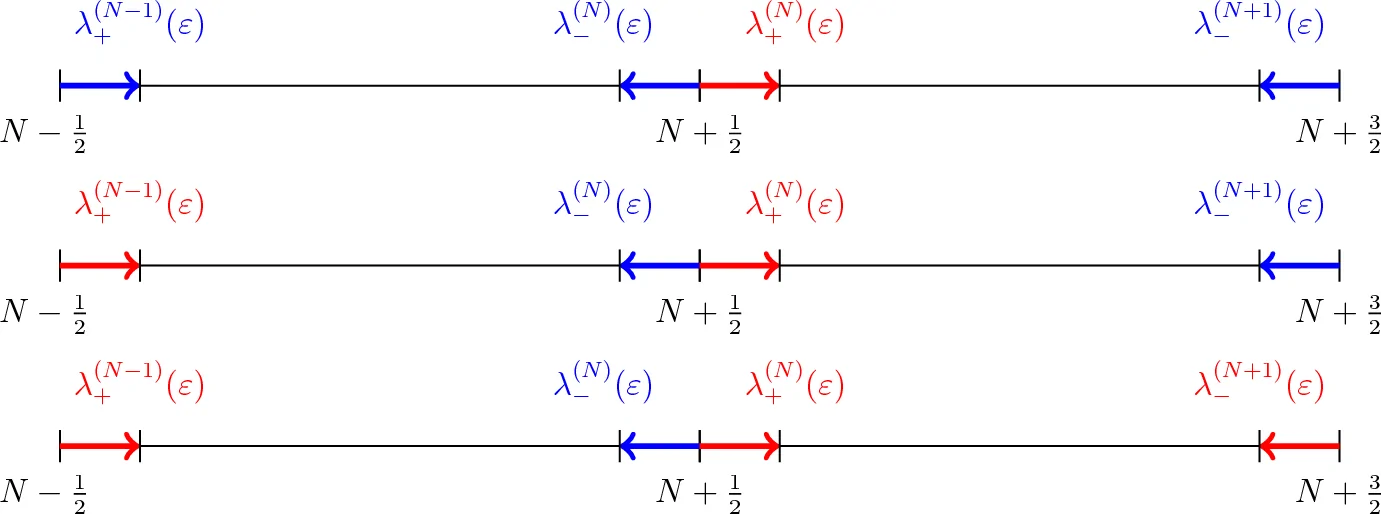
Possible distribution of eigenvalues in the non-degenerate case: different colors denote different parities. The arrows denote the splitting of the eigenvalues for the perturbed model respect to the harmonic oscillator eigenvalues

This is equivalent to the rule that, in each parity sector, two empty adjacent intervals and two doubly occupied adjacent intervals are forbidden; see [1, 8, 15]. (To verify the conjecture one can just look at the behaviour of the eigenvalues in two adjacent intervals [N-1/2,N+1/2) and [N+1/2,N+3/2). In Fig. 1 the three cases in which the conjecture fails are shown.) In the perturbative regime the assertion follows from the directions in which neighboring eigenvalue branches move and from Proposition 3.4 (see also Fig. 2 for the distribution of eigenvalues in the non-degenerate case).

#### Corollary 3.6

(Generic couplings) Assume $2\alpha ^2\notin \mathsf Z$ and $\gamma _1=-\gamma _2$. For every *K* there exists $c_K>0$ such that Braak’s conjecture holds in all intervals [N+1/2,N+3/2) with N≤K and $0<|\varepsilon |<c_K$.

#### Proof sketch

In the genuine Rabi case $\beta _1=0$, and the first-order coefficients in (19) have opposite signs. Hence one branch born at N+1/2 moves upward and the other downward. Each interval [N+1/2,N+3/2) consequently receives the upward branch from level *N* and the downward branch from level N+1. Their parities are $\sigma _N$ and $-\sigma _{N+1}$. Applying the same description to the adjacent interval gives the parity-resolved bounds in Conjecture 3.5. See [12, Section 5] for the endpoint bookkeeping. □

### How the interval count works

We now describe the local counting argument behind Corollary 3.6. Assume ε>0; the case ε<0 is obtained by reversing the labels. Since $\beta _1=0$,

$$ \lambda _{N,+}(\varepsilon )>N+\frac{1}{2}, \qquad \lambda _{N,-}(\varepsilon )<N+\frac{1}{2} $$

for all levels in the chosen finite window and sufficiently small ε. Therefore

$$ \lambda _{N,+}(\varepsilon ),\ \lambda _{N+1,-}(\varepsilon ) \in [N+1/2,N+3/2). $$

The same statement at the next level gives

$$ \lambda _{N+1,+}(\varepsilon ),\ \lambda _{N+2,-}(\varepsilon ) \in [N+3/2,N+5/2). $$

By Proposition 3.4, the four parities are

$$ \sigma _N,\quad -\sigma _{N+1},\quad \sigma _{N+1},\quad -\sigma _{N+2}. $$

In particular, the two branches issued from the intermediate level N+1 occur in adjacent intervals and have opposite parity. Thus each parity contributes at least one and at most three eigenvalues to the union of two adjacent intervals, while no single interval contains more than two eigenvalues of a fixed parity. Together, these remarks give the two inequalities stated in Conjecture 3.5.

## Exceptional couplings and higher-order splitting

Assume now that $2\alpha ^2\in \mathsf Z$ and define

20 $$\begin{aligned} \mathsf K_\alpha =\{N\in \mathbb {Z}_+:L_N(2\alpha ^2)=0\}. \end{aligned}$$

For $N\in \mathsf K_\alpha$, the first Rellich matrix is scalar, so first-order perturbation theory does not distinguish the two branches. The interlacing of Laguerre zeros implies

21 $$\begin{aligned} N\in \mathsf K_\alpha \quad \Longrightarrow \quad N-1,N+1\notin \mathsf K_\alpha . \end{aligned}$$

This isolation of exceptional levels is central in the interval-counting argument.

### Parity without a nondegeneracy assumption

Even if the arguments in Sect. 3.2 cannot be implemented anymore, analytic spectral projections retain the parity structure. Let

22 $$\begin{aligned} P_N(\varepsilon )=-\frac{1}{2\pi i}\int _\Gamma (\widehat{Q}_{R,\varepsilon } -z)^{-1}\,dz, \end{aligned}$$

where Γ encloses only the double eigenvalue N+1/2 of $\widehat{Q}_{R,0}$. The projection is analytic and has rank two for small ε.

At ε=0, choose $\zeta _\pm =(\zeta _\pm ^1,\zeta _\pm ^2)^T$ satisfying

23 $$\begin{aligned} \zeta _\pm ^1=\pm (-1)^N\zeta _\pm ^2. \end{aligned}$$

Then $U_0T^*(\phi _N\otimes \zeta _\pm )$ have opposite parity. Applying $P_N(\varepsilon )$ and normalizing gives two analytic vectors in $\operatorname {Ran}P_N(\varepsilon )$ of opposite parity.

#### Proposition 4.1

(Parity in an exceptional cluster) For every exceptional level $N\in \mathsf K_\alpha$ and sufficiently small |ε|, the two-dimensional spectral subspace $\operatorname {Ran}P_N(\varepsilon )$ contains a nonzero vector of each parity. Consequently, the two analytic eigenvalue branches emerging from N+1/2 can be labeled so that their eigenspaces meet opposite parity subspaces.

#### Proof

Let

$$ v_{N,\pm }(0)=U_0T^*(\phi _N\otimes \zeta _\pm ). $$

The condition (23), together with $\phi _N(-x)=(-1)^N\phi _N(x)$, shows directly that $v_{N,\pm }(0)\in \boldsymbol{H}_\pm$. Set

$$ \widetilde{v}_{N,\pm }(\varepsilon ) =P_N(\varepsilon )v_{N,\pm }(0). $$

Since $[Q_{R,\varepsilon },\Pi ]=0$, the resolvent and the contour integral (22) commute with Π. Hence $\widetilde{v}_{N,\pm }(\varepsilon )\in \boldsymbol{H}_\pm$. Furthermore,

$$ \Vert \widetilde{v}_{N,\pm }(\varepsilon )-v_{N,\pm }(0)\Vert _0 \le \Vert P_N(\varepsilon )-P_N(0)\Vert \,\Vert v_{N,\pm }(0)\Vert _0\rightarrow 0,\,\,\,\varepsilon \rightarrow 0, $$

so neither vector vanishes for small ε. After normalization, we obtain one analytic vector of each parity in $\operatorname {Ran}P_N(\varepsilon )$. Since this range has dimension two, its intersections with $\boldsymbol{H}_+$ and $\boldsymbol{H}_-$ are both one-dimensional. Every eigenvalue subspace inside the cluster is parity invariant, which gives the stated labeling of the two analytic branches. □

**Fig. 3:**
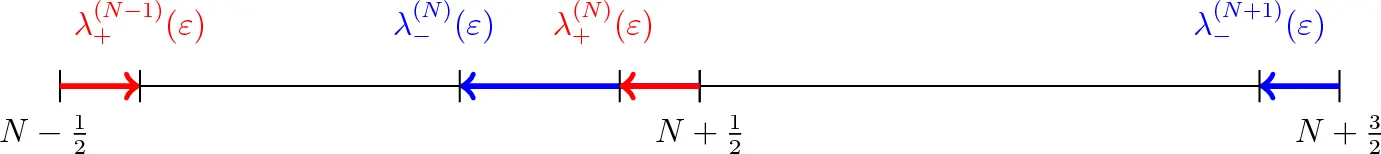
Distribution of eigenvalues in the degenerate case: different colors denote different parities. The arrows denote the splitting of the eigenvalues for the perturbed model respect to the harmonic oscillator eigenvalues

Combining Proposition 4.1, the generic analysis of the neighboring levels, and the interlacing property (21) (see also Fig. 3 for the distribution of the eigenvalues in the degenerate case), one obtains the finite-window theorem announced in the introduction.

#### Theorem 4.2

(Braak’s conjecture for arbitrary couplings) For every $k_0\in \mathbb {N}$ there exists $\varepsilon _0>0$ such that, for every $N\le k_0$ and every $0<|\varepsilon |\le \varepsilon _0$, Braak’s conjecture holds in [N+1/2,N+3/2). Moreover,

$$ \#\bigl (\operatorname {Spec}\widehat{Q}_{R,\varepsilon } \cap [N+1/2,N+3/2)\bigr )\in \{1,2,3\}. $$

#### Proof sketch

The only configuration not covered by Corollary 3.6 occurs when one or both endpoint levels of two adjacent unit intervals are exceptional. By (21), exceptional indices are never adjacent, so the intermediate level is non-degenerate and contributes one upward and one downward simple branch with known parities. Proposition 4.1 says that every exceptional two-dimensional cluster contributes one state of each parity, independently of the order at which the eigenvalues separate. In the worst case, an interval may contain both states from an exceptional cluster and one state from a neighboring generic level, yielding at most three total eigenvalues but at most two in either parity. Applying the same argument to the adjacent interval excludes two consecutive empty or doubly occupied parity sectors. The complete case division is written out in [12, Section 6]. □

#### Remark 4.3

The perturbation parameter in the genuine Rabi model is the atomic gap: replacing Δ by $\varepsilon \Delta$ makes Theorem 4.2 a small-gap statement. The length of the controlled spectral window is arbitrary but fixed since the admissible size of ε may shrink as the window is enlarged.

## Multi-level Rabi systems and Weyl asymptotics

### Three configurations

Let N≥3 and n=N-1. Write $E_{jk}$ for the N×N matrix which is 1 in the (*j*, *k*) entry and 0 otherwise and

$$ p_2^{\mathrm w}(x,D)=\frac{|D|^2+|x|^2}{2} $$

for the *n*-dimensional oscillator. The following matrix Hamiltonians model an *N*-level atom coupled to *n* cavity modes.

In the Ξ-configuration,

24 $$\begin{aligned} A_\Xi =p_2^{\mathrm w}I_N +\sum _{k=1}^{N-1}\alpha _kx_k(E_{k,k+1}+E_{k+1,k}) +\sum _{k=1}^{N-1}\gamma _kE_{k+1,k+1}. \end{aligned}$$

In the ⋀-configuration,

25 $$\begin{aligned} A_\wedge =p_2^{\mathrm w}I_N +\sum _{k=1}^{N-1}\alpha _kx_k(E_{k,N}+E_{N,k}) +\sum _{k=1}^{N-1}\gamma _kE_{k+1,k+1}. \end{aligned}$$

In the ⋁-configuration,

26 $$\begin{aligned} A_\vee =p_2^{\mathrm w}I_N +\sum _{k=1}^{N-1}\alpha _kx_k(E_{1,k+1}+E_{k+1,1}) +\sum _{k=1}^{N-1}\gamma _kE_{k+1,k+1}. \end{aligned}$$

The principal symbol is scalar and elliptic and the obstruction is at order 1: the coupling matrix can have crossings that prevent a smooth global diagonalization in the Shubin class. Thus the systems need not belong directly to the SMGES class of [10].

This failure is visible already in the simplest coupling blocks. A real coordinate $x_k$ vanishes on a codimension-one hypersurface, and the corresponding eigenvalue branches meet there. Replacing $x_k$ by the complex phase-space coordinate $x_k+i\varepsilon \xi _k$ moves the zero set to

$$ x_k=\xi _k=0. $$

For all modes simultaneously, the common zero is only the phase-space origin, which is excluded in the homogeneous diagonalization problem. This is why an arbitrarily small momentum component removes the global crossing obstruction.

The remedy, when N=3 and n=2, is to insert a small momentum component. For instance, in the Ξ case set

27 $$\begin{aligned} b_1(X)=\sum _{k=1}^{N-1}\alpha _k \bigl ((-i\xi _k)E_{k,k+1}+i\xi _kE_{k+1,k}\bigr ). \end{aligned}$$

Then

28 $$\begin{aligned} x_k\pm i\varepsilon \xi _k \end{aligned}$$

replace the real coupling coordinate. For every ε>0, the corresponding semiprincipal matrix has the regularity and spectral separation needed for smooth diagonalization away from X=0. Analogous choices work for (25) and (26).

#### Proposition 5.1

(Microlocal regularization) Let N=3 and n=2. For each of the three configurations (24)–(26) there is a self-adjoint homogeneous order-one matrix $b_1$ such that $a_1+\varepsilon b_1$ is smoothly unitarily diagonalizable on $\mathbb {R}^{2n}\setminus \{0\}$ for every ε>0. The eigenvalues can be arranged into globally ordered blocks, and therefore $a+\varepsilon b_1$ is an SMGES.

#### Idea of the proof

In the Ξ configuration, introduce $\psi _{k,\varepsilon }=(x_k+i\varepsilon \xi _k)/\sqrt{2}$. The coupling matrix becomes the weighted adjacency matrix of a chain with complex edge weights $\psi _{k,\varepsilon }$. Away from X=0, not all edge weights vanish. Their phases can be removed by a smooth diagonal unitary, leaving positive moduli $|\psi _{k,\varepsilon }|$. The resulting real symmetric Jacobi matrix has simple ordered spectrum when the couplings are nonzero. The ⋀ and ⋁ configurations are treated by the same phase removal on the star-shaped coupling graph, followed by a block decomposition if eigenvalue multiplicities persist. The detailed matrix diagonalizations are given in [12, Section 7]. □

### Comparison with a regularized system

We now prove Theorem 1.2. Let

$$ A=a^{\mathrm w}(x,D),\qquad B_1=b_1^{\mathrm w}(x,D),\qquad A_\varepsilon =A+\varepsilon B_1. $$

The domain is $B^2(\mathbb {R}^n;\mathbb {C}^N)$ for both *A* and $A_\varepsilon$. Since $B_1$ has order one and *A* has order two, the operator

$$ A^{-1/4}B_1A^{-1/4} $$

has order zero and is bounded on $L^2$. Therefore, with

$$ C=\Vert A^{-1/4}B_1A^{-1/4}\Vert _{L^2\rightarrow L^2}, $$

we have

29 $$\begin{aligned} |((A_\varepsilon -A)u,u)_0| \le \varepsilon C(A^{1/2}u,u)_0, \qquad u\in B^2. \end{aligned}$$

Equivalently,

30 $$\begin{aligned} (Au,u)_0-\varepsilon C(A^{1/2}u,u)_0 \le (A_\varepsilon u,u)_0 \le (Au,u)_0+\varepsilon C(A^{1/2}u,u)_0. \end{aligned}$$

Let $\lambda _j$ and $\lambda _{j,\varepsilon }$ be the eigenvalues of *A* and $A_\varepsilon$, repeated with multiplicity. The minimax principle gives

31 $$\begin{aligned} \lambda _j-\varepsilon C\sqrt{\lambda _j} \le \lambda _{j,\varepsilon } \le \lambda _j+\varepsilon C\sqrt{\lambda _j}. \end{aligned}$$

Define the inverse comparison maps

32 $$\begin{aligned} \nu _{\varepsilon ,\pm }^{-1}(\lambda ) =\lambda \pm \varepsilon C\sqrt{\lambda }. \end{aligned}$$

For ε small enough, they are increasing on the positive spectral half-line, and (31) becomes the counting-function sandwich

33 $$\begin{aligned} \mathsf N_{A_\varepsilon }(\nu _{\varepsilon ,-}^{-1}(\lambda )) \le \mathsf N_A(\lambda ) \le \mathsf N_{A_\varepsilon }(\nu _{\varepsilon ,+}^{-1}(\lambda )). \end{aligned}$$

For fixed ε>0, the operator $A_\varepsilon$ is SMGES. The two-term Weyl law of [10] yields

34 $$\begin{aligned} \mathsf N_{A_\varepsilon }(\Lambda ) =(2\pi )^{-n}\bigg (&N\Lambda ^n\int _{p_2\le 1}dX\nonumber \\&-\Lambda ^{n-1/2}\int _{p_2=1}\operatorname {Tr}(a_1+\varepsilon b_1) \frac{ds}{|\nabla p_2|}\bigg ) +O_\varepsilon (\Lambda ^{n-1}). \end{aligned}$$

We want to replace Λ by (32),

35 $$\begin{aligned} (\lambda \pm \varepsilon C\sqrt{\lambda })^n&=\lambda ^n\pm n\varepsilon C\lambda ^{n-1/2}+O_\varepsilon (\lambda ^{n-1}),\end{aligned}$$

36 $$\begin{aligned} (\lambda \pm \varepsilon C\sqrt{\lambda })^{n-1/2}&=\lambda ^{n-1/2}+O_\varepsilon (\lambda ^{n-1}). \end{aligned}$$

After division by $\lambda ^{n-1/2}$, the remainder in (34) tends to zero. The two sides of (33) thus give upper and lower bounds for

$$ \lambda ^{-(n-1/2)}\left( \mathsf N_A(\lambda )-(2\pi )^{-n}N\lambda ^n\int _{p_2\le 1}dX \right) . $$

The bounds differ from

$$ -(2\pi )^{-n}\int _{p_2=1}\operatorname {Tr}(a_1)\frac{ds}{|\nabla p_2|} $$

by terms of size O(ε). Letting first $\lambda \rightarrow \infty$ and then $\varepsilon \downarrow 0$ proves (2).

#### Remark 5.2

The order of the regularizing perturbation explains the precision of the argument. Since $B_1$ has order one relative to an order-two operator, the eigenvalue displacement is $O(\sqrt{\lambda })$, exactly the scale of the second Weyl term. The comparison captures that term but does not retain a smaller remainder uniformly as $\varepsilon \rightarrow 0$.

### Application to the Rabi configurations when N=3 and n=2

For the Ξ-model, the choice (27) gives

$$ a_1+\varepsilon b_1 =\sum _{k=1}^{2}\alpha _k \left( (x_k-i\varepsilon \xi _k)E_{k,k+1} +(x_k+i\varepsilon \xi _k)E_{k+1,k}\right) . $$

Writing $\psi _{k,\varepsilon }=(x_k+i\varepsilon \xi _k)/\sqrt{2}$ exhibits the same creation-annihilation geometry as in the Jaynes–Cummings family and gives the required smooth block diagonalization for ε>0. For the ⋀- and ⋁-models, one inserts the same momentum component in the corresponding matrix entries. Therefore Theorem 1.2 applies to all three configurations.

In the displayed Rabi examples the order-one matrix is off-diagonal, so $\operatorname {Tr}(a_1)=0$. Consequently, the coefficient of $\lambda ^{3/2}$ vanishes and

37 $$\begin{aligned} \mathsf N_A(\lambda ) =(2\pi )^{-2}N\lambda ^2\int _{p_2\le 1}dX +o(\lambda ^{3/2}). \end{aligned}$$

The theorem is stated with the trace term because it applies without change to more general semiregular systems with scalar harmonic oscillator principal part and nonzero diagonal semiprincipal contribution.

Since $\{p_2\le 1\}$ is the ball of radius $\sqrt{2}$ in $\mathbb {R}^{4}$,

$$ \int _{p_2\le 1}dX=\frac{(2\pi )^2}{2}. $$

Thus the leading term in (37) takes the especially simple form

38 $$\begin{aligned} \mathsf N_A(\lambda )=\frac{3}{2}\lambda ^2 +o(\lambda ^{3/2}). \end{aligned}$$

For N=2, the asymptotics given by Theorem 1.2 reproduces the doubled one-dimensional oscillator leading coefficient. For N=3, it gives the phase-space volume law for the three-level models.

## Concluding remarks

The perturbative analysis of the one-mode model and the high-energy analysis of its multi-mode generalizations use different scales, but they rely on the same structural idea: transform a non-diagonal matrix Hamiltonian into a setting in which the scalar harmonic oscillator geometry becomes available.

At fixed energy, metaplectic translations reduce the problem to a doubled oscillator with a bounded nonlocal perturbation. Hermite functions then turn translations into explicit finite sums, and the exceptional set is given by Laguerre zeros. At high energy, a small momentum regularization converts a non-diagonalizable semiprincipal symbol into a globally diagonalizable one. The Weyl asymptotics of the regularized family can then be transferred back by a variational comparison.

Several questions remain open. The finite-window result does not provide a perturbation size uniform as the energy tends to infinity. Such a statement would require quantitative control of Laguerre overlaps and of higher-order effective matrices. In the multi-mode setting, it would be useful to determine whether one can bypass the auxiliary regularization and construct a calculus adapted directly to the crossing geometry of the Rabi semiprincipal symbol. These problems lie naturally at the interface of Fourier analysis, microlocal diagonalization, and spectral theory.

## Data Availability

No data sets were generated or analyzed in this work.
